# Persistence of a stage-structured food-web

**DOI:** 10.1038/s41598-017-11686-z

**Published:** 2017-09-08

**Authors:** Akihiko Mougi

**Affiliations:** Department of Biological Science, Faculty of Life and Environmental Science, Shimane University, 1060 Nishikawatsu-cho, Matsue, 690-8504 Japan

## Abstract

Contrary to a theoretical prediction, natural communities comprise many interacting species, thereby developing complex ecosystems. Earlier theoretical studies assumed that each component species within an ecological network has a simple life history, despite the fact that the interaction partners of many species, such as their predators and resources, change during the developmental stages. This poses an open question on the effect of life history complexity on the dynamics of communities. Here using a food web model, I showed that species with a stage-structured life cycle greatly changes the relationship between community complexity and persistence. Without stage-structured species, an increase in species diversity and interaction links decreases the community persistence, whereas in the presence of stage-structured species, community complexity can increase the community persistence. Therefore, life history complexity may be a key element of biodiversity that is self-maintaining.

## Introduction

Determining the mechanism by which biodiversity is maintained is a challenging subject in ecology. The observations of nature reveal a positive relationship between complexity and stability in natural ecosystems^1^, contrary to a theoretical prediction^2^. Over the last four decades, bridging the gap between theory and observations in nature has been attempted to understand the mechanism by which biodiversity is maintained^3, 4^. A number of theoretical studies have proposed realistic interaction network structures, such as topology or interaction strength, as candidates for the mechanism that maintain complex communities^5–8^.

However, most theoretical studies considered a community in which the component species have a simple life cycle, despite the fact that in reality, communities comprise species with various life history types^9–12^. Some animal has a simple life cycle; however, most animals experience an ontogenetic niche shift, in other words, an ecological change in diet or interaction partners during their lifespan^9, 10^. A large effect of ontogenetic niche shift on the community dynamics has been shown by many theoretical studies which assume only small and simple systems^11–13^.

A recent theoretical study showed a large effect of ontogenetic niche shift on the robustness of a large and complex community, suggesting that ontogenetic niche shifts reduce stability^14^. This cannot explain species diversity in nature. However, the structural robustness^15^, used as an index of stability (susceptibility to secondary extinctions), does not consider the population dynamics and their feedbacks, leaving an unanswered question of how ontogenetic niche shifts affect the consequences of population dynamics, thereby leading to the classical complexity–stability debate. On the other hands, classical May’s approach^2^ is also based on the extreme assumption of local stability of equilibrium population dynamics. To overcome such problems, I adopt food-web persistence as a measure of stability, which is defined as the proportion of persistent species during a sufficiently long period (Methods).

Here I developed a theoretical food web comprising species with simple or stage-structured life cycles in varying proportions to reveal the role of life history diversity in the maintenance of complex communities. To examine the role of only ontogenetic niche shifts on food web persistence, a random food web with *N* species and interaction links determined by the proportion of connected pairs *P* was used (Methods). The food webs comprised species with a simple life cycle and species with a stage-structured life cycle. By changing the proportion of species with stage-structured life cycle within a food web *p*_c_, I examined the effect of life cycle diversity on food web persistence.

## Results

**Figure 1:**
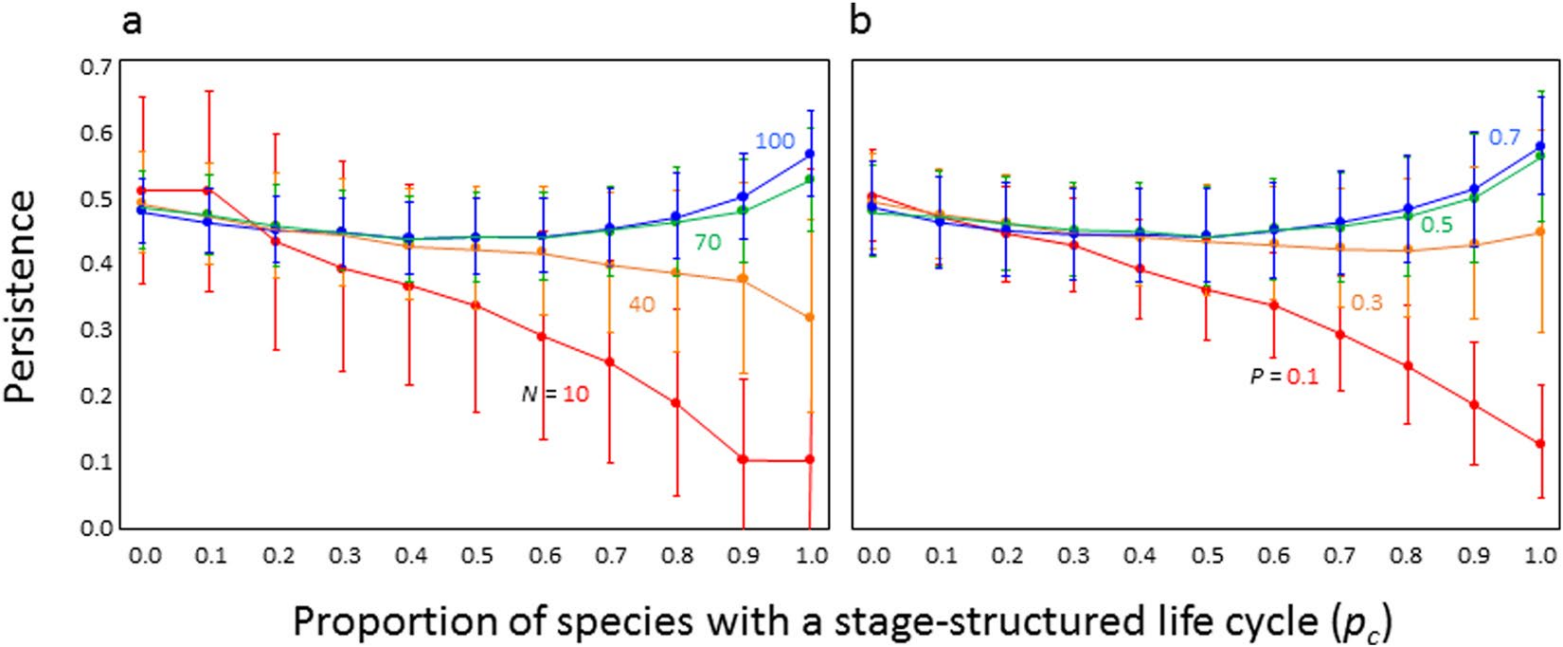
Relationship between the proportion of species with a stage-structured life cycle (*p*_*c*_) and community persistence. (**a**) Effect of species richness *N*. I assumed that the proportion of connected pairs (*P*) was equal to 0.3. (**b**) Effect of *P*. I assumed that *N = *50. Each point and line indicates mean and error bars, respectively. See details of parameter values in the Methods section.

The mixing of different life cycle types within food webs has a major effect on the persistence (Fig. 1). Introduction of species with a stage-structured life cycle into food webs may decrease or increase the community persistence depending on the food web complexity (*N*, *P*). In simpler food webs (lower species richness or less connected pairs), stage-structured species has a negative effect on community persistence, whereas in more complex food webs (higher species richness or more connected pairs), it has a positive effect on community persistence. In more complex food webs, the persistence has two peaks in pure simple life and stage-structured food webs. These results suggest that the lifecycle types have a qualitative difference on the effects of the relationship between complexity and stability.

**Figure 2:**
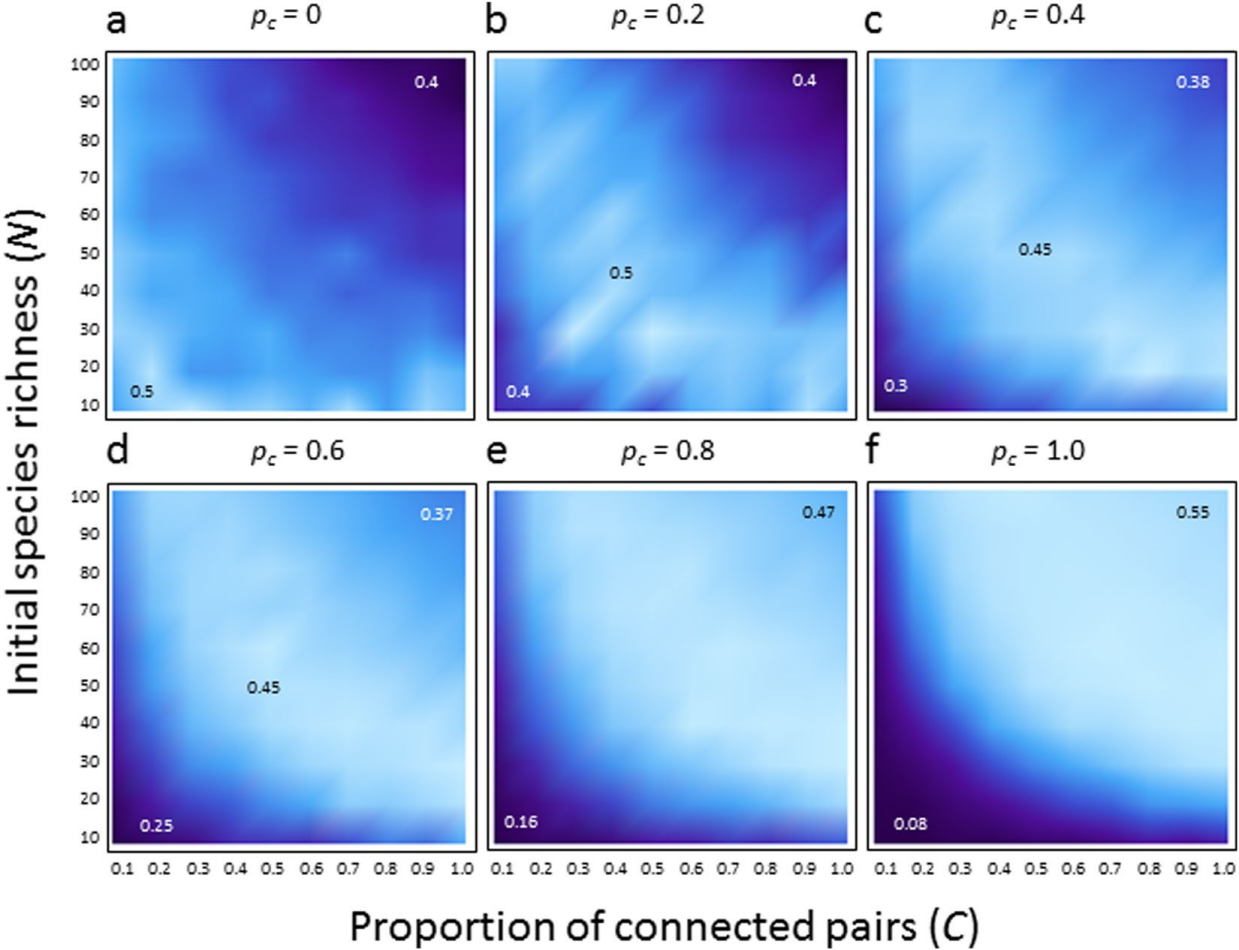
Complexity–persistence relationships with varying proportion of species with a stage-structured life cycle (*p*_*c*_). Contour indicates the levels of community persistence (shown by numbers). Lighter shade indicates higher stability. Parameter values are same as those in Fig. 1.

The life cycle types greatly affect the complexity–stability relationship, on-going debate in ecology^4^. In food webs with only simple lifecycle, increased complexity destabilized the food webs (Fig. 2a), congruent with earlier theoretical studies^2^. However, with a moderate mixing of different life cycle types, complexity affected the food web persistence non-monotonically. Food webs with an average level of complexity had a peak in persistence (Fig. 2b–d). Furthermore, in food webs more skewed to a stage-structured life cycle, positive complexity–persistence relationships tended to be observed (Fig. 2e,f). The positive effect of complexity on persistence did not depend on the presence of interactions between stages (Figs S1, S2), the differences in network topologies between stages (Fig. S3), and the network structures (Fig. S4). However, the result crucially depended on maturation probability (maturation rate/decline rates in juveniles) (Fig. S5). The maturation probability tends to become lower by higher juvenile death rates and/or weaker interaction coefficients. The results show that the positive complexity–persistence is likely to appear when the maturation probability is lower (Fig. S5). This result suggests that the food webs that comprised many species with ontogenetic niche shifts can be stabilized, rather than destabilized by community complexity, particularly when maturation probability is not high.

## Discussion

Contrary to earlier studies that highlighted the network structures of ecological communities^16–24^, the present study suggests the importance of life history, an essential feature in organisms, on the balance of nature^11–14^. Furthermore, the result clearly showed that ontogenetic niche shift is a key for the positive relationship between complexity and persistence, contrary to a previous theory in which population dynamics are not assumed^14^. This contradictory predictions suggest that population dynamics is crucial for understanding the stability of stage-structured food webs.

The mechanism of positive complexity–persistence would be related to positive feedback from one stage to another, which can decrease persistence in simple food web models^13^. If juveniles are not likely to mature, adults also lose their resources, resulting in decreases in the juveniles’ resources. Indeed, the present study suggests that this negative cycle is likely to occur when maturation probability is low. However, increasing community complexity can weaken such negative cycles because multiple resources increase maturation probability. In addition, the lower maturation probability, which can create a positive effect of complexity on persistence, is caused by higher juvenile death rates and/or lower interaction coefficients. These conditions would be natural in real food webs, given the apparent vulnerability of juveniles and weak interactions in food webs, as reported by empirical studies^25–27^.

A traditional approach has viewed a node of the interaction network as one species^2, 4^; however, this approach may not be able to capture the dynamics of ecological communities. The extinction of a species is not just the loss of a species, but the loss of a part of the community of species with life history diversities, which may lead to unexpected consequences. Communities with different proportions of life history types may respond differently to species loss. Notably, communities skewed toward complex life history types show a strong positive complexity–persistence relationship, suggesting that species loss further decreases stability, leading to cascading species loss. Such extinction may be caused not only by losses of species but also by complex life histories within a community.

Ecological community diversity or complexity is assumed to have evolved from simple communities over time. In addition, life history diversities of organisms would similarly have developed from simple life histories. The development or increasing complexity in communities may require the life history complexity in the community members, or life histories and communities may evolve together. Consequently, the field of conservation biology should pay attention not only to the species itself but also to the life history types of the species^14, 28^. In addition, the theory provides a testable prediction on the relationship between the composition of life history types and community size. Larger communities are expected to include a higher proportion of species with complex life histories.

There are challenging tasks that need to be addressed in the future. To compare the present study with previous studies, I used a traditional stability index and a standard stage-structured model. However, there are diverse types of stability measures^29^ and more realistic continuous ontogenetic development models^30^. In addition, The present structured model does not persist without external input. This is simply because the species cannot increase if there are no autotrophic resource species in the system. A realistic model with autotroph and heterotroph is needed to further understanding of structured food webs. Hence, it leaves an unanswered question of whether a positive complexity-stability effect caused by ontogenetic niche shifts is robust compared with other or more appropriate stability measures and more realistic models.

The present study sheds new light on the concept of biodiversity. Earlier theories proposed that species diversity, one of the key elements of biodiversity, as well as other elements, such as genetic^6^, population^31^, and interaction-type diversity^8^, can synergistically contribute to maintain the biodiversity itself. The traditional approach has focused on the roles of interaction network structures such as topology and interaction strengths for community stability without considering such diverse types of complexity^3–5, 7^. Biodiversity inherently complicates network structures, suggesting a limitation of the traditional network approach^32^. Life history diversity may also be one of the key elements of a complex biodiversity network. Diverse species with stage-structured life cycles which experience ontogenetic niche shift, utilize not only different interaction partners but also different spatial environments, depending on the life stages, suggesting a link between life history and ecosystem diversities or biotic and abiotic environment diversity.

## Methods

Consider a food web where *N* species may interact through prey–predator relationships. In the food web, a proportion of the species members has a simple life history without a stage structure, whereas the rest have a complex life history with a stage structure. The two life history stages, juvenile and adult, were considered. In the model, there were no interactions between stages, and network topologies of each stage were independently and randomly constructed or were not the same between stages (see Figs S1–S3 for cases where these assumptions are relaxed). The interaction pairs are randomly determined in each life stage. To perform the direct test of ontogenetic niche shifts on the community stability, I assumed a random food web^33^ (see Fig. S4 in a cascade network) and linear functional response, similar to previous studies^2, 24^. The population dynamics of species *i* with a simple life history is described as follows:

1 $$\frac{d{X}_{i}^{S}}{dt}={X}_{i}^{S}({r}_{i}-{s}_{i}{X}_{i}^{S}+{g}_{i}\sum _{j\in resources}{a}_{ij}{X}_{j}-\sum _{j\in consumers}{a}_{ji}{X}_{j}),$$

where *X*_*i*_^*S*^ is the abundance of species *i*, *r*_*i*_ is the intrinsic rate of change in species *i*, *s*_*i*_ is the density-dependent self-regulation, *a*_*ij*_ is the interaction coefficient between species *i* and *j*, and *g*_*i*_ is the conversion efficiency, which relates the birth rate to its resource consumption. In contrast, the population dynamics of species *i* with a stage structure is described as follows:

2a $$\frac{d{X}_{i}^{J}}{dt}=B{X}_{i}^{A}-{X}_{i}^{J}\sum _{j\in consumers}{a}_{ji}{X}_{j}-M{X}_{i}^{J}-{D}_{J}{X}_{i}^{J},$$

2b $$\frac{d{X}_{i}^{A}}{dt}=M{X}_{i}^{J}-{X}_{i}^{A}\sum _{j\in consumers}{a}_{ji}{X}_{j}-{D}_{A}{X}_{i}^{A},$$

where *X*_*i*_^*J*^ and *X*_*i*_^*A*^ are the juvenile and adult abundances of species *i*, respectively, *B* is the birth rate, *M* is the maturation rate of the juvenile, *D*_*J*_ is the death rate of the juvenile, and *D*_*A*_ is the death rate of the adult. I assumed the simple forms of density-dependent self-regulations in the death rates. In this model, constant birth rates are also necessary for allowing populations to grow at least (otherwise all species always become extinct). The forms of each life-history processes are as follows: *B = b*_*i*_ + ${g}_{i}\sum _{j\in resources}{a}_{ij}{X}_{j}$, *M* = ${m}_{i}\sum _{j\in resources}{a}_{ij}{X}_{j}$, *D*_*J*_ = *d*_*i*_^*J*^ − $(1-{m}_{i})\,\sum _{j\in resources}{a}_{ij}{X}_{j}$  +  *s*_*i*_^*J*^ *X*_*i*_^*J*^, and *D*_*A*_ = *d*_*i*_^*A*^ − $(1-{g}_{i})\,\sum _{j\in resources}{a}_{ij}{X}_{j}$ + *s*_*i*_^*A*^ *X*_*i*_^*A*^, where *b*_*i*_ is the basal constant birth rate of species *i*, *m*_*i*_ is the maturation rate of species *i*, *d*_*i*_^*j*^ (*j = J* or *A*) is the constant death rate of species *i* in each stage, and *s*_*i*_^*j*^ (*j = J* or *A*) is the self-regulation of species *i* in each stage. The second term in death rates represents the effects of reducing starvation due to resource consumptions^13^. I also tested two other types of models: (1) a model with a density dependence in birth and maturation and (2) a model with a self-regulation in death processes: (1) *B* = (*b*_*i*_ + ${g}_{i}\sum _{j\in resources}{a}_{ij}{X}_{j}$)/(1 + *X*_*i*_^*A*^), *M* = (${m}_{i}\sum _{j\in resources}{a}_{ij}{X}_{j}$)/(1 + *X*_*i*_^*J*^), *D*_*J*_ = *d*_*i*_^*J*^, and *D*_*A*_ = *d*_*i*_^*A*^; and (2) *B* = *b*_*i*_ + ${g}_{i}\sum _{j=1,\,j\ne i}^{N}{a}_{ij}{X}_{j}$, *M* = ${m}_{i}\sum _{j=1,\,j\ne i}^{N}{a}_{ij}{X}_{j}$, *D*_*J*_ = *d*_*i*_^*J*^ + *s*_*i*_^*J*^ *X*_*i*_^*J*^, *D*_*A*_ = *d*_*i*_^*A*^  +    *s*_*i*_^*A*^ *X*_*i*_^*A*^. The units of parameters are per unit time, except for *g* that is a unitless parameter. I confirmed that these models show qualitatively similar behaviors (Fig. S6).

I defined the proportion of connected pairs *P* as the proportion of realized interaction links *L* in the possible maximum interaction links *L*_*max*_ [ = *N* (*N* − 1)/2) of a given network model (*L = PL*_*max*_). I controlled the proportion of species with a stage-structured life cycle within a food web, *p*_c_, to examine the effects of life-history diversity on population dynamics. Simple life species can randomly interact with other simple life species, adults of stage-structured species, and/or juveniles of stage-structured species.

Here I assume *s*_*i*_ and *s*_*i*_^*j*^ (*j = J* or *A*) are constant (*s*_*i*_ *= s*_*i*_^*j*^ = 1) following May^2^. Without self-regulations, unbounded population growths are likely to occur and the communities do not persist. Parameters *b*_*i*_, *g*_*i*_ (<1) *m*_*i*_ (<1) and initial abundances are randomly chosen from a uniform distribution between (0 and 1). *r*_*i*_, *a*_*ij*_ and *d*_*i*_^*j*^ are randomly chosen from a uniform distribution between (−1 and 1), (0 and 10^−1^) and (0 and 10^−1^), respectively. Obviously, the conversion efficiency and maturation rate should be less than 1 in the biological sense. The maximum values of other parameters (or ranges) are minimum requisite for the persistence of communities (e.g. larger values of interaction coefficients cause destabilization and make impossible to persist and larger death rates also make impossible to persist). Non-large values of constant birth rates are set to allow the inherent local community dynamics. Note that more realistic situation, higher death rates of juvenile than adult, is likely to make positive complexity effect to persistence (Fig. S5). The default values of parameters are summarized in Table S1. See Fig. S5 for details of parameter dependence on the results. I also tested the effects of relaxing uniform distributions. By using Beta distribution, *β*(*α*, *β*), we can study the effects of varying the distribution of parameter values. The result shows that the main result does not change qualitatively (Fig. S7).

Stability was defined as “persistence” estimated as the mean proportion of species that survive after a sufficiently long period of population dynamics across 1,000 sample communities^34^. The population dynamics is calculated by Mathematica (a sample code is shown in Supplementary information).

## Electronic supplementary material

Supplemental information
