# Supplementary material for: Persistence of a stage-structured food-web
Source: Sci Rep. 2017 Sep 8;7:11055. doi: 10.1038/s41598-017-11686-z (PMC5591249; doi:10.1038/s41598-017-11686-z)
Supplement: Supplementary file 1 — Supplemental information [file 41598_2017_11686_MOESM1_ESM.pdf]

Supplementary Information

Persistence of a stage-structured food-web

A. Mougi

Supplemental figures

Fig. S1. Relationship between the proportion of foraging links to adults by juveniles (p_{ja}) and community persistence. (a) Effect of species richness *N*. I assumed proportion of connected pairs (*P*) = 0.3. (b) Effect of proportion of *P*. I assumed *N* = 50. I considered food webs without simple life cycles ($p_c = 1.0$). In addition to the interactions within the life stage, I considered the following interactions between stages: adults feed on juveniles and/or juveniles feed on adults. The proportion of foraging links to adults by juveniles and that of foraging links to juveniles by adults are defined as p_{ja} and $1 - p_{ja}$, respectively. See details of parameter values in the Methods section.

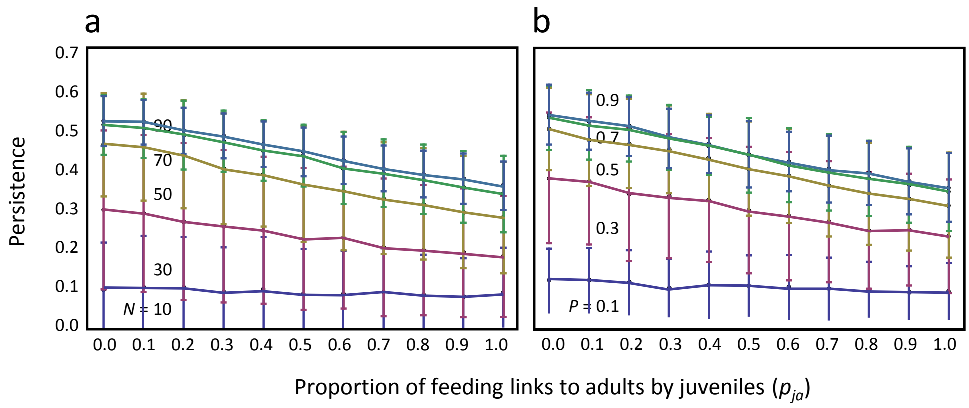

Fig. S2. Complexity–stability relationships with varying proportions of foraging links to adults by juveniles (p_{ja}). Other information is the same as that in Fig. S1.

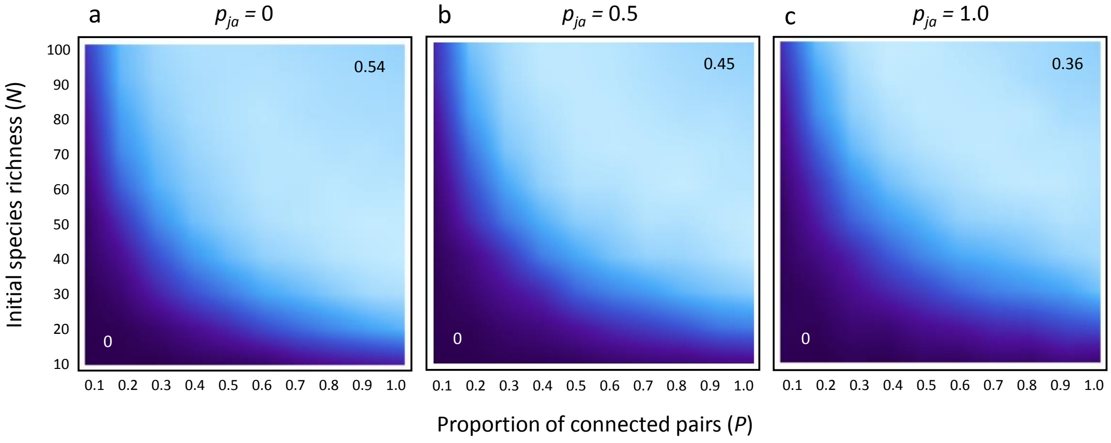

Fig. S3. Complexity–stability relationships without cross feedings between life stages. (a) Food webs with the same network topologies between stages. (b) Food webs with different network topologies between stages. I considered food webs without a simple life cycle (p_c = 1.0).

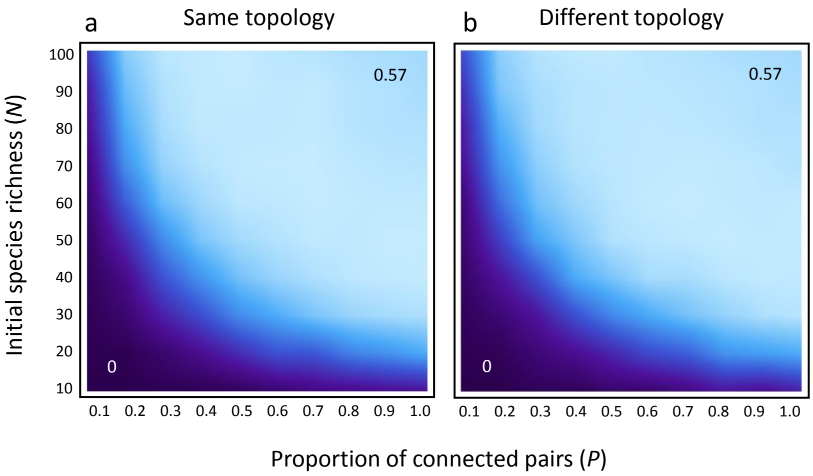

Fig. S4. Complexity–stability relationships in a cascade model. In the cascade model, for each pair of species, i, j = 1,…, n with $i < j$, species i never consumes species j, whereas species j may consume species i. I considered food webs without a simple life cycle (p_c = 1.0). Maximum values of d_J and d_A in a uniform distribution are assumed to be 0.05 and 0.01, respectively.

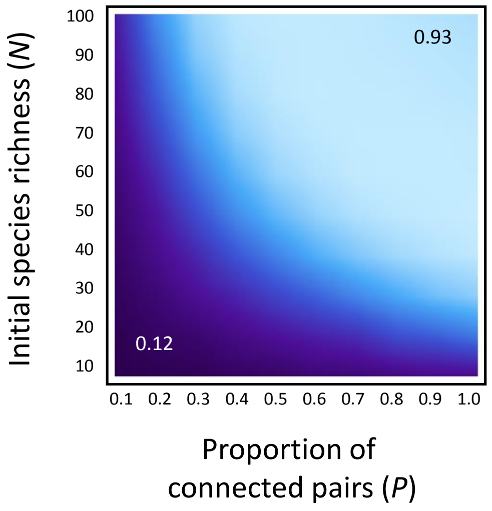

Fig. S5. Complexity–stability relationships with varying parameters that affects maturation probabilities. (a-c) Effects of maximum value of maturation rates in a uniform distribution, m_{max}. (d-f) Effects of maximum value of juvenile death rates in a uniform distribution, d_{Jmax}. I assumed maximum value of interaction coefficients in a uniform distribution, a_{max} = 0.1. (g-i) Effects of maximum value of interaction coefficients in a uniform distribution, a_{max}. I assumed d_{Jmax} = 0.05. I considered food webs without a simple life cycle (p_c = 1.0).

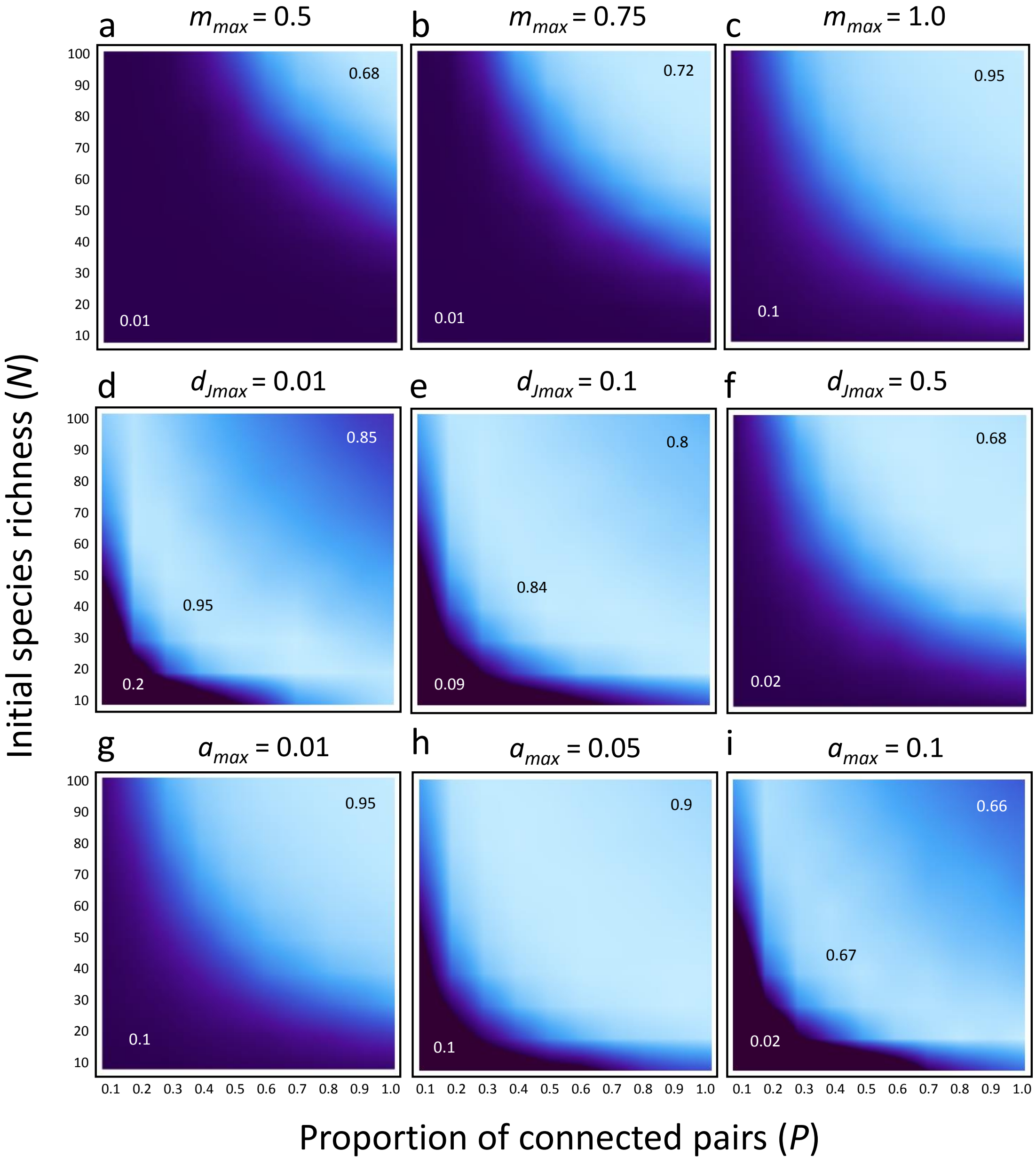

Fig. S6. Complexity–stability relationships in alternative models with only stage-structured species (p_c =1.0) shown in Methods. (a) Model (1). I assumed a_{max} = 0.01. (b) Model (2). I assumed a_{max} = 0.02. s_i^J and s_i^A are randomly chosen from a uniform distribution between (0 and 1). Other parameter values are same with those used in main model.

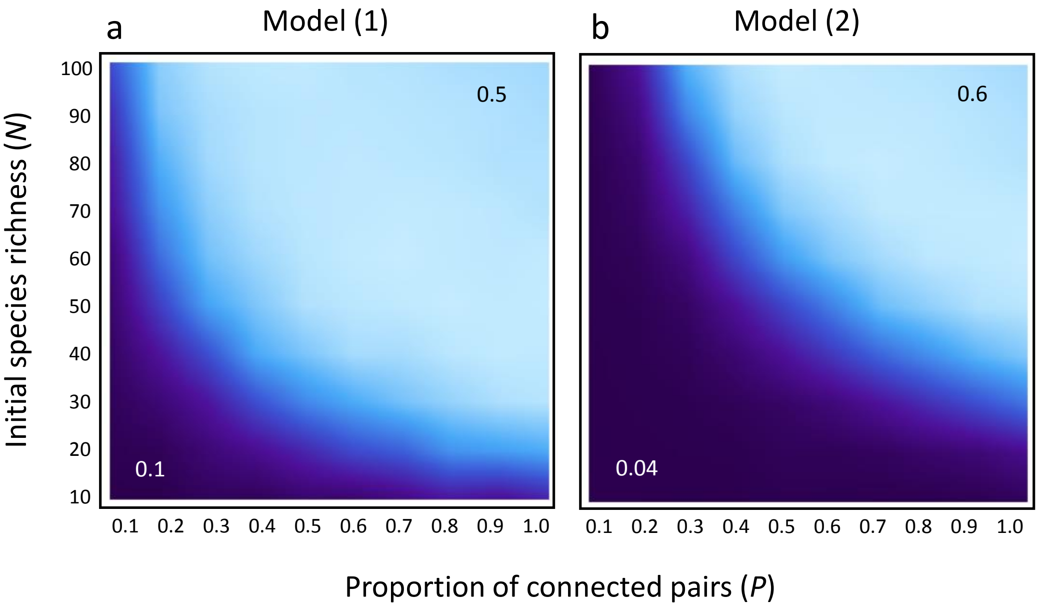

Fig. S7. Effects of varying distribution of parameters on the relationship between complexity and persistence in a stage-structured model. (a) Effects of variance (symmetrical distribution is assumed, α = β). (b) Effects of asymmetrical skewness. I assumed α = 1.0. (c) Effects of asymmetrical skewness. I assumed β= 1.0. N = 50, m_i is randomly chosen from a uniform distribution between (0 and 0.5). Other parameter values are same with those used in the main text.

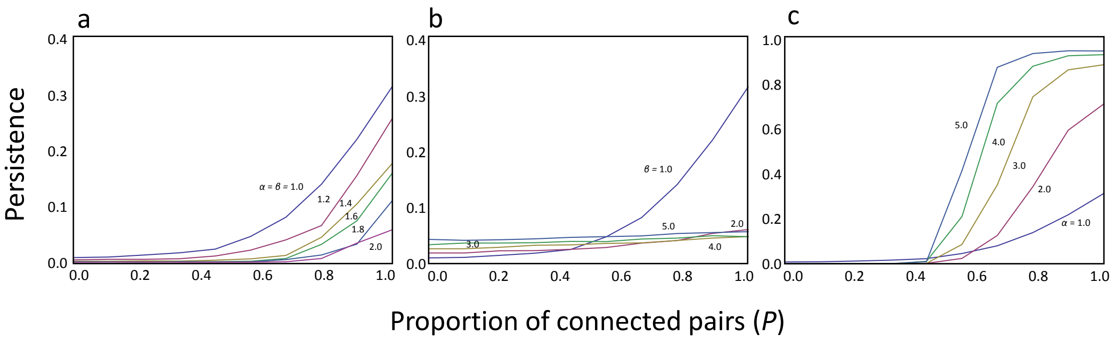

Supplemental table

Table S1. Default values of parameters.

Default values of parameters

Parameter	Parameter range or value
r_i	(-1~1)
s_i	1
s_i^j	1
b_i	(0~1)
g_i	(0~1)
m_i	(0~1)
d_i^j	(0~0.1)
a_{ij}	(0~0.1)

Sample code of Mathematica

Population dynamics of communities with a stage-structured species are repronounced by the following sample code:

```
s=40;c=0.5;
A=0.1;

B= RandomReal[{0,1},s];
b= RandomReal[{0,1},s];
γ= RandomReal[{0,1},s];
G= 0 RandomReal[{0,1},s];

ϕ=0.1 RandomReal[{0,1},s];
d=0.1 RandomReal[{0,1},s];

∈j=1;
∈a=1;

aJJ=A RandomReal[{0,1},{s,s}];
aJA=A RandomReal[{0,1},{s,s}];
aAJ=A    RandomReal[{0,1},{s,s}];
aAA=A    RandomReal[{0,1},{s,s}];

xini=RandomReal[1,2 s];

time=3000;

popsizeJ=Table[Table[xJ[i][n],{i,s}],{s}];
popsizeA=Table[Table[xA[i][n],{i,s}],{s}];

popsize={};AppendTo[popsize,Transpose[popsizeJ]];AppendTo[popsize,Transpose[po
psizeA]];
popsize=Flatten[popsize,1];
popsize=Flatten[Transpose[popsize]];
popsize=Flatten[AppendTo[popsize,popsize]];
popsize=Partition[popsize,2 s];
```

```
NumPred=Round[0.5*s(s-1)*c/2];
NumPrey=NumPred;
list=Flatten[{Table[-1,{NumPred}],Table[1,{NumPrey}],Table[0,{0.5*s(s-1)-
NumPred-NumPrey}]}];

sample=RandomSample[Range[Length[list]],Length[list]];
sample=Table[list[[sample[[i]]]],{i,1,Length[list]}];
plus=Reverse[Table[i,{i,2,s-1}]];
plus=Accumulate[plus]+Table[i,{i,1,Length[plus]}];
Do[sample=Insert[sample,Table[0,{1+i}],plus[[i]]],{i,1,Length[plus]}]
sample=PrependTo[sample,0];
sample=Join[sample,Table[0,{s}]];
sample=Flatten[sample];
sample=Partition[sample,s];

pred1=ReplacePart[sample,Position[sample,1]→0];
pred2=ReplacePart[sample,Position[sample,-1]→0];
pred3=ReplacePart[pred1,Position[pred1,-1]→1]+Transpose[pred2];
pred=-1*Transpose[pred1]*γ+pred2*γ;
ichi=ReplacePart[sample,Position[sample,-1]→1];
ichiaJJ=ichi*aJJ+Transpose[ichi*aJJ];
intermatr=sample-Transpose[sample];

interactionJJ=ichiaJJ*intermatr*popsizeJ*(-pred+pred3);

sample1=RandomSample[Range[Length[list]],Length[list]];
sample1=Table[list[[sample1[[i]]]],{i,1,Length[list]}];

Do[sample1=Insert[sample1,Table[0,{1+i}],plus[[i]]],{i,1,Length[plus]}]
sample1=PrependTo[sample1,0];
sample1=Join[sample1,Table[0,{s}]];
sample1=Flatten[sample1];
sample1=Partition[sample1,s];

pred11=ReplacePart[sample1,Position[sample1,1]→0];
```

```
pred12=ReplacePart[sample1,Position[sample1,-1]→0];
ichi2=pred12-Transpose[pred11];
ichiaAA=ichi2*aAA;
interactionAA=-ichiaAA*popsizeA;

interactionnokori=Table[0,{i,1,s},{j,1,s}];

birth=Transpose[-interactionAA]*popsizeA*b;
birth=Total[Transpose[birth]];

birth=birth*IdentityMatrix[s];
birth=birth+B popsizeA ;
birth=birth*IdentityMatrix[s];
birth=birth/popsizeJ    ;

survivea=Transpose[-interactionAA]*popsizeA*(1-b);
survivea=Total[Transpose[survivea]];
survivea=survivea*IdentityMatrix[s];

survivea=survivea/popsizeA;

mature=(-Transpose[pred1]+pred2)*interactionJJ;
mature=Total[Transpose[mature]];
mature=mature*IdentityMatrix[s];
consmature=G IdentityMatrix[s];
mature=mature-consmature;

mature=mature popsizeJ/popsizeA;

survivej=-1*Transpose[pred1]*(1-γ)+pred2*(1-γ);
survivej=ichiaJJ*intermatr*popsizeJ*(-survivej);
survivej=Total[Transpose[survivej]];
survivej=survivej*IdentityMatrix[s];

Jdeath=(-ϕ-∈j popsizeJ)    IdentityMatrix[s]-survivej;
Jdeath=Jdeath-consmature;
```

```
Adeath=(-d-∈a popsizeA ) IdentityMatrix[s]+survivea;
zero=Table[0,{i,s}];
Do[Adeath=Insert[Adeath,zero,2 i+1],{i,0,s}]
Adeath=Partition[Flatten[Adeath],2 s];
Do[Jdeath=Insert[Jdeath,zero,2 i],{i,1,s}]
Jdeath=Partition[Flatten[Jdeath],2 s];

death=AppendTo[Jdeath,Adeath];
death=Partition[Flatten[death],2 s];

upperrightmatrix=birth;
lowerleftmatrix=(interactionnokori-mature);

Do[upperrightmatrix=Insert[upperrightmatrix,zero,2 i+1],{i,0,s}]
upperrightmatrix=Partition[Flatten[upperrightmatrix],2 s];
Do[interactionJJ=Insert[interactionJJ,zero,2 i],{i,1,s}]
interactionJJ=Partition[Flatten[interactionJJ],2 s];

uppermatrix=interactionJJ+upperrightmatrix;

Do[interactionAA=Insert[interactionAA,zero,2 i+1],{i,0,s}]
interactionAA=Partition[Flatten[interactionAA],2 s];
Do[lowerleftmatrix=Insert[lowerleftmatrix,zero,2 i],{i,1,s}]
lowerleftmatrix=Partition[Flatten[lowerleftmatrix],2 s];
lowermatrix=interactionAA+lowerleftmatrix;

interactionmatrix=AppendTo[uppermatrix,lowermatrix];
interactionmatrix=Partition[Flatten[interactionmatrix],2 s];
interactionmatrix=interactionmatrix+death;

interaction=interactionmatrix popsize[[1]];

left1=Table[xJ[i]'[n],{i,s}];
left2=Table[xA[i]'[n],{i,s}];
left=Join[left1,left2];
```

```
equation=Table[left[[i]]==Total[interaction[[i]]],{i,1,2 s}];

inipopsizeJ=Table[xJ[i][0],{i,s}];
inipopsizeA=Table[xA[i][0],{i,s}];
inipopsize=Join[inipopsizeJ,inipopsizeA];

initial=Table[inipopsize[[i]]==xini[[i]],{i,1,2 s}];

equplusinitial=Join[equation,initial];

variable1=Table[xJ[i],{i,1,s}];
variable2=Table[xA[i],{i,1,s}];
variable=Join[variable1,variable2];

kai=NDSolve[equplusinitial,variable,{n,0,time}];

listlast=Flatten[Table[Evaluate[popsizeJ[[1]]+popsizeA[[1]]/.kai],{n,time,time}]];
listpersist={};
Do[If[listlast[[i]]>10^-3,AppendTo[listpersist,listlast[[i]]]],{i,1,s}];

Plot[Evaluate[{popsizeJ[[1]]+popsizeA[[1]]}/.kai],{n,0,time},PlotRange→{0,1}]
Length[listpersist]/s//N
```
